# Factors influencing language development in children with hearing loss: a qualitative study of parental attitudes and behaviors

**DOI:** 10.3389/fpsyg.2026.1807202

**Published:** 2026-05-21

**Authors:** Nazmiye Evra Günhan Şenol, Bahtiyar Çelikgün, Melike Nur Dede, Okan Kılınç

**Affiliations:** 1 Department of Speech and Language Therapy, School of Health Sciences, Istanbul Medipol University, Istanbul, Türkiye; 2 Department of Audiology, School of Health Sciences, Istanbul Medipol University, Istanbul, Türkiye; 3 Department of Audiology, Graduate School of Health Sciences, İstanbul Medipol University, Istanbul, Türkiye

**Keywords:** hearing loss, language development, parental attitude, pediatric hearing loss, reflexive thematic analysis

## Abstract

**Objective:**

This study aimed to explore how parents of children with hearing loss understand, emotionally process, and engage with auditory rehabilitation, with particular attention to differences between parents of children with age-appropriate versus delayed language development.

**Methods:**

A qualitative design was employed using focus group interviews with parents of children with hearing loss. Participants were grouped according to their children’s language development outcomes. Data were collected through a semi-structured interview guide and analyzed using reflexive thematic analysis following an iterative and inductive approach. Reporting was guided by the Consolidated Criteria for Reporting Qualitative Research (COREQ).

**Results:**

The analysis identified a set of interrelated themes reflecting parental engagement across the rehabilitation process, including emotional responses following diagnosis, approaches to managing rehabilitation, perceptions of child development, everyday communication practices, adaptation to hearing devices, and family dynamics. While several experiences were shared across groups, parents of children with age-appropriate language development more consistently described early acceptance, sustained engagement, and integration of communication strategies into daily routines. In contrast, parents of children with delayed language development more frequently reported prolonged emotional distress, inconsistencies in engagement, and challenges in maintaining rehabilitation-related practices.

**Conclusion:**

Parental engagement in pediatric auditory rehabilitation is shaped not only by access to services, but also by parental meaning-making, emotional readiness, and the ability to translate clinical recommendations into everyday family practices. These findings highlight the importance of integrating structured, family-centered counseling approaches that address emotional processes, expectation alignment, and practical skill-building to support sustained caregiver engagement and optimize rehabilitation outcomes.

## 1 Introduction

Hearing loss is one of the most common congenital conditions worldwide, with an estimated prevalence of approximately 1.5 per 1,000 live births, and it remains a major global public health concern due to its potential long-term developmental consequences when not identified and managed early (Morton and Nance, 2006). According to the World Health Organization (WHO), more than 5% of the global population experiences disabling hearing loss, and a substantial proportion of affected children require timely access to auditory assistive technologies and structured rehabilitation services to support optimal communication outcomes (WHO, 2024). As with other developmental conditions, early diagnosis followed by appropriate intervention plays a critical role in minimizing adverse effects on speech, language, and psychosocial development.

In pediatric hearing loss, rehabilitation outcomes are influenced not only by clinical variables related to the child, such as the degree, type, and laterality of hearing loss, age at diagnosis, and timing of amplification or cochlear implantation, but also by family-related factors (Salamatmanesh, 2021). Previous studies have emphasized the importance of parental involvement, consistent device use, and the quality of parent–child interaction in supporting language development in children who use hearing aids or cochlear implants (Çelikgün and Akdaş, 2021). In addition, family dynamics and communication patterns have been associated with broader developmental and adaptive outcomes in children with hearing loss (Dunn, 1997).

In Türkiye, nationwide newborn hearing screening programs and referral systems have substantially improved the early identification of pediatric hearing loss over the past two decades. Following newborn hearing screening, children with suspected hearing loss are generally referred to diagnostic, amplification, and rehabilitation services within the healthcare system. However, variability may still occur in how consistently families engage with follow-up and rehabilitation processes after diagnosis. Previous studies conducted in Türkiye have suggested that factors such as family engagement with follow-up services, consistency in rehabilitation participation, healthcare navigation, access to information, and attendance at multiple referral centers may influence rehabilitation trajectories despite the availability of screening and referral systems (Turan and Baş, 2019).

While several quantitative studies have explored predictors of hearing device use and language outcomes, such approaches may be limited in their ability to capture the complexity of parental experiences and everyday practices that shape the rehabilitation process. Parental attitudes toward the diagnosis, emotional responses, coping strategies, and interpretations of professional guidance are inherently experiential and context-dependent phenomena. These processes often involve meaning-making and appraisal, which are difficult to fully represent through standardized quantitative measures alone (Creswell and Poth, 2016; Lazarus and Folkman, 1984). Qualitative research methods are therefore particularly well suited to exploring how parents understand and navigate the challenges associated with raising a child with hearing loss.

It is widely acknowledged that effective family involvement is associated with more favorable language outcomes in children with hearing loss (Ching, 2015). However, there remains limited qualitative evidence examining how parental attitudes, thoughts, and behaviors differ according to children’s observed language development trajectories. In particular, little is known about how parents of children with age-appropriate language development may differ from those whose children demonstrate delayed language development in terms of coping with the diagnosis, managing the rehabilitation process, and supporting communication in everyday life.

Qualitative inquiry offers a means of addressing these gaps by allowing for an in-depth exploration of parents’ narratives and the meanings they attribute to their experiences (Creswell and Poth, 2016). By focusing on parents’ own accounts, qualitative approaches can illuminate mechanisms underlying engagement with rehabilitation, consistency of device use, and patterns of parent–child communication that may not be readily apparent in quantitative designs. Such insights are essential for informing family-centered models of auditory rehabilitation and for tailoring professional support to the diverse needs of families.

Within the context of patient education and counseling, parental engagement is not solely determined by access to information, but also by how parents interpret, internalize, and implement professional guidance in everyday interactions with their child. Effective counseling in pediatric hearing loss therefore requires more than the provision of technical knowledge; it involves supporting parents in translating recommendations into meaningful and sustainable practices within the family context. Understanding these processes is essential for developing counseling strategies that enhance caregiver engagement and long-term adherence to rehabilitation.

The aim of the present study was therefore to qualitatively explore and compare the attitudes, thoughts, and behaviors of parents of children with hearing loss whose language development was aligned with their chronological age and those whose children demonstrated delayed language development. Through focus group discussions, this study sought to address the following research question: “How do parental attitudes, perceptions, and everyday practices related to auditory rehabilitation differ between families of children with age-appropriate versus delayed language development?” By addressing this question, the study aims to contribute to a more nuanced understanding of family-related factors in pediatric auditory rehabilitation and to inform clinical practices aimed at supporting both children with hearing loss and their families.

## 2 Methods

### 2.1 Study design and epistemological framework

This study employed a qualitative research design informed by an interpretivist epistemological perspective. Within interpretivist paradigms, reality is understood as socially constructed, and meaning is generated through individuals’ interactions with their social and cultural environments rather than existing as an objective, measurable entity (Creswell and Poth, 2016). From this standpoint, parents’ attitudes, thoughts, and behaviors related to their children’s auditory rehabilitation are conceptualized as context-dependent interpretations shaped by lived experience.

A reflexive thematic analysis (RTA) approach, as articulated by Braun and Clarke (2006, 2021), was selected as the primary analytic method. Reflexive thematic analysis is particularly appropriate for qualitative studies that seek to explore patterns of meaning across a dataset while acknowledging the active role of the researcher in the analytic process. Unlike positivist or codebook-driven approaches, RTA does not assume themes to be inherent within the data; rather, themes are generated through a recursive and reflexive engagement between the researcher and the dataset (Braun and Clarke, 2021).

The selection of RTA was guided by the aims and design of the study. First, the research sought to explore how parents make sense of their experiences following a diagnosis of hearing loss and throughout the auditory rehabilitation process, rather than to test predefined hypotheses or generate a formal theory. Second, the use of focus group interviews required an analytic approach capable of capturing both individual perspectives and meanings co-constructed through group interaction. Finally, reflexive thematic analysis offers sufficient flexibility to accommodate interpretative depth while remaining methodologically rigorous, making it well suited for exploratory qualitative research in health-related contexts (Braun and Clarke, 2006).

The analytic orientation of the study was primarily inductive and semantic. Themes were developed from participants’ explicit accounts rather than being imposed *a priori* from existing theoretical frameworks. Nevertheless, analytic interpretation was informed by the researchers’ disciplinary knowledge in audiology and auditory rehabilitation, consistent with reflexive qualitative practice. This positioning recognizes researcher subjectivity as an analytic resource rather than a source of bias, provided that reflexive engagement is made transparent throughout the research process (Braun and Clarke, 2021).

### 2.2 Sampling strategy and participants

A purposive sampling strategy was employed to recruit participants with direct and sustained experience relevant to the research question. In qualitative research, purposive sampling aims to achieve depth of understanding by selecting information-rich cases based on predefined criteria rather than statistical representativeness (Creswell and Poth, 2016).

Participants were recruited from parents and primary caregivers of children with hearing loss who were receiving auditory rehabilitation services at a speech and language therapy clinic. Participants were selected based on the following criteria: (1) being a primary caregiver of a child with hearing loss, (2) the child actively using hearing aids or cochlear implants, (3) regular participation in auditory rehabilitation sessions, and (4) willingness to share experiences in a group discussion setting. Inclusion criteria further required that the child be between 3 and 7 years of age and attend auditory rehabilitation sessions on a regular basis. Children with additional diagnoses beyond hearing loss or documented intellectual disability were excluded in order to reduce confounding developmental factors and maintain analytic focus on hearing-related rehabilitation experiences.

Participants were identified through the rehabilitation center’s records according to these eligibility criteria and were invited to participate on a voluntary basis. No participant selection was made based on anticipated responses, attitudes, or rehabilitation outcomes. Recruitment was conducted independently of the families’ ongoing clinical care, and no therapeutic or supervisory relationship existed between the research team and the participants.

To provide contextual differentiation within the sample, children’s language development was assessed using the Turkish version of the Preschool Language Scale-Fifth Edition (PLS-5) (Sahli and Belgin, 2017). The PLS-5 was not employed as an analytic instrument within the qualitative analysis; rather, it functioned as a descriptive and organizational framework to support the formation of focus groups and contextualize parental narratives. Based on PLS-5 classifications, parents were grouped according to whether their children’s language development was broadly aligned with chronological age or reflected delayed language development. To minimize the risk of shaping or constraining parental accounts, specific test scores and group classifications were not disclosed to participants during the data collection process.

A total of 10 parents participated in the study and were distributed across two focus groups. Although the numerical sample size was small, it was considered appropriate for the aims of this qualitative inquiry. In line with contemporary qualitative methodological guidance, sample adequacy was evaluated in terms of information power rather than data saturation (Malterud et al., 2016). The specificity of the research question, the relative homogeneity of the rehabilitation context, and the richness of the focus group discussions supported the adequacy of the sample for in-depth thematic analysis.

Participants were recruited from the same rehabilitation center and shared a broadly similar clinical and urban service context. This approach was intended to reduce excessive contextual variability and allow for a more focused exploration of differences in parental meaning-making, attitudes, and everyday practices related to children’s language development. Demographic characteristics of participating parents and children are presented in Tables 1 and 2.

**Table 1:** Descriptive information about children and their parents in the age-appropriate language development group (group A).

Parents					Children							
Participants	Age	Parents	Education	Job	Number of siblings	Gender	Age	Age at diagnosis (months)	Hearing device	Degree of hearing loss	Hearing loss unilateral/bilateral	PLS-5 scores
HK	50	Father	University	Employee	2	Female	4	0–12	Hearing aids	Severe SNHL	Bilateral	64
EÇ	26	Mother	University	—	2	Female	3	0–12	Cochlear imp.	Profound SNHL	Bilateral	61
ÇA	29	Mother	University	Designer	2	Male	5	12–36	Cochlear imp.	Profound SNHL	Bilateral	60
ŞG	34	Mother	High School	—	2	Female	3	0–12	Hearing aids	Severe SNHL	Bilateral	62
Zİ	26	Mother	High School	—	2	Male	4	12–36	Cochlear imp.	Profound SNHL	Bilateral	64

**Table 2:** Descriptive information about children and their parents in the delayed language development group (group B).

Parents					Children							
Participants	Age	Parents	Education	Job	Number of siblings	Gender	Age	Age at diagnosis (months)	Hearing device	Degree of hearing loss	Hearing loss unilateral/bilateral	PLS-5 scores
ZŞ	54	Grandmother	Primary s.	—	2	Female	6	0–12	Cochlear imp.	Profound SNHL	Bilateral	48
TA	52	Mother	Primary s.	—	3	Male	6	0–12	Cochlear imp.	Profound SNHL	Bilateral	44
YÇ	27	Mother	High school	—	2	Female	7	+36	Hearing aids	Severe SNHL	Bilateral	41
ŞH	39	Mother	University	Teacher	2	Male	6	24–36	Hearing aids	Severe SNHL	Bilateral	50
HC	31	Mother	Primary s.	—	2	Male	5	0–12	Hearing aids	Severe SNHL	Bilateral	40

### 2.3 Data collection

Data were collected through focus group interviews, a method well suited to exploring shared experiences, collective meanings, and interactive dynamics among participants within a common context. Focus groups were selected to facilitate discussion among parents and to allow participants to reflect on, elaborate, and respond to one another’s experiences related to the auditory rehabilitation process.

Two focus group interviews were conducted, each comprising parents of children with hearing loss grouped according to their children’s language development profiles. One focus group included parents of children whose language development was broadly aligned with their chronological age, while the second group included parents of children with delayed language development. The focus group discussions were conducted on two separate days, with the group comprising parents of children with delayed language development interviewed first, followed by the group of parents of children with age-appropriate language development 5 days later.

All interviews were conducted in a meeting room at the rehabilitation center where the children were receiving auditory rehabilitation services. This setting was selected to ensure participant convenience and familiarity. The focus group discussions were conducted in Turkish, the participants’ native language, to allow for rich and nuanced expression of experiences and perceptions. Each session was facilitated by two moderators, both of whom were graduate students in audiology and co-authors of the study. Prior to data collection, the moderators received training in qualitative interviewing and focus group facilitation under the supervision of senior researchers experienced in auditory rehabilitation and qualitative research methods. The moderators had academic backgrounds in audiology and previous clinical observation experience in pediatric auditory rehabilitation settings; however, they were not involved in the clinical management or therapeutic decision-making processes of the participating children. No prior personal relationship existed between the moderators and the participants before recruitment, apart from limited professional familiarity within the rehabilitation setting. This positioning was considered beneficial in facilitating communication while minimizing potential power imbalance during the discussions. In addition to the moderators, two audiology interns attended each session as observers and took field notes to capture non-verbal interactions, group dynamics, and contextual details that could not be fully conveyed through audio recordings alone.

The moderators were not involved in the clinical management or rehabilitation of the participating children and had no prior relationship with the families. This separation was intentionally maintained to minimize potential response bias related to perceived expectations or authority.

In addition, participants were not informed about the specific grouping of their children (age-appropriate vs. delayed language development) in order to avoid influencing their responses.

To further reduce social desirability bias, participants were explicitly informed that there were no right or wrong answers and that their responses would not affect their child’s care in any way. Moderators adopted a neutral, non-evaluative stance throughout the interviews and avoided leading questions or affirming responses.

A semi-structured interview guide was used to facilitate the discussions. The guide was developed by the authors based on a review of the relevant literature and consultation with experts in the field of audiology and auditory rehabilitation. It consisted of six open-ended questions organized around four broad topic areas: experiences related to auditory rehabilitation, the amplification or implantation process, the role of the social environment, and parents’ psychological and emotional responses throughout the rehabilitation journey. The semi-structured format allowed moderators to ensure consistency across focus groups while also providing flexibility to pursue emergent topics raised by participants. The core open-ended questions included:

1. Can you describe how you felt when you first learned about your child’s hearing loss and how you managed this process?
2. How did people around you respond to your child’s hearing loss and hearing device use, and how did you react to these responses?
3. What role do you play in your child’s auditory rehabilitation process? For example, how do you support your child’s communication at home or in everyday environments, and do you participate in rehabilitation sessions?
4. How do you communicate with your child, and how does your child communicate with you? For example, does your child primarily use verbal communication, gestures, or other communication strategies?
5. How did you manage the process of helping your child adapt to hearing aids or cochlear implants? How long did this process take, and how did you respond when your child resisted device use?
6. How do you monitor your child’s hearing device or cochlear implant use throughout the day? Can you describe your daily routines related to device use, maintenance, or monitoring?

Each focus group discussion was audio-recorded with the informed consent of all participants. The duration of the focus group sessions was 52 min for the group of parents of children with delayed language development and 66 min for the group of parents of children with age-appropriate language development. Audio recordings were transcribed verbatim following each session. To enhance data accuracy and completeness, transcripts were reviewed alongside moderators’ and observers’ field notes. All identifying information was removed during the transcription process to protect participant confidentiality.

### 2.4 Data analysis

Data were analyzed using reflexive thematic analysis, following the approach proposed by Braun and Clarke (2006, 2021). This analytic method was applied to explore patterns of meaning in parents’ accounts regarding their experiences with their children’s auditory rehabilitation and language development. Although parents were grouped based on their children’s language development outcomes, these groupings were used to contextualize parental narratives rather than to support direct analytical comparisons. The analysis focused on identifying patterns of meaning across accounts, with attention to variation rather than causal or statistical differences between groups.

Analysis began after the completion of data collection and transcription. Audio recordings from both focus group interviews were transcribed verbatim in Turkish. The transcripts were read repeatedly by the research team to achieve familiarity with the content and to gain an overall sense of participants’ narratives. During this stage, the researchers reviewed the transcripts alongside field notes taken by moderators and observers in order to retain contextual information related to group dynamics and interactional features of the discussions.

Initial coding was conducted inductively across the entire dataset. Segments of text that reflected parents’ attitudes, emotional responses, interpretations of the diagnosis process, approaches to rehabilitation, and everyday communication practices were coded. Coding was not restricted to predefined categories; instead, codes were generated directly from participants’ accounts and remained open to modification throughout the analytic process.

Following initial coding, codes were examined and actively organized into candidate themes based on shared patterns of meaning. This process involved moving beyond surface-level descriptions to consider how parents made sense of their experiences and how these meanings were articulated within and across the two focus groups. At this stage, particular attention was paid to patterns that appeared to characterize parents of children with age-appropriate language development and those whose children demonstrated delayed language development, while avoiding formal comparative claims that would exceed the interpretative scope of the qualitative design.

Candidate themes were then reviewed and refined through iterative engagement with the data. The research team revisited the original transcripts to ensure that each theme was internally coherent, clearly distinguishable from other themes, and adequately supported by data extracts. Some themes were merged, refined, or redefined during this process to improve conceptual clarity and analytic precision.

Once the thematic structure was finalized, themes were defined and named to capture their central organizing concepts. Representative quotations were selected to illustrate each theme and to demonstrate how the analytic interpretations were grounded in participants’ accounts. Throughout the analysis, the researchers engaged in reflexive discussion to consider how their professional backgrounds in audiology and auditory rehabilitation may have influenced analytic decisions and interpretations.

Participant quotations were translated from Turkish into English by the research team and reviewed by the primary researcher to preserve conceptual meaning.

### 2.5 Reflexivity and trustworthiness

#### 2.5.1 Reflexivity

Reflexivity was embedded throughout the analytic process. The research team had formal training in qualitative research methodologies obtained through doctoral education in Applied Language and Speech Sciences, Audiology, and Speech and Language Disorders. The primary researcher was not involved as a moderator during the focus group interviews but contributed to the development of the interview guide, supervised the analytic process, and reviewed the translation of participant quotations from Turkish to English to support conceptual accuracy. The absence of a prior relationship between moderators and participants, together with the use of neutral interviewing practices and semi-structured questioning, was considered important in minimizing potential influence on participant responses. The interdisciplinary backgrounds of the researchers informed sensitivity to patterns of meaning, communication practices, and parental narratives during data interpretation. Throughout the analysis, reflexive discussions within the research team were used to critically examine how prior disciplinary knowledge and assumptions may have influenced analytic decisions and interpretations.

#### 2.5.2 Trustworthiness

Trustworthiness was addressed through established qualitative criteria. Credibility was supported through verbatim transcription, prolonged engagement with the data, and the use of illustrative participant quotations. Dependability was enhanced by providing a transparent account of data collection and analytic procedures. Confirmability was supported through reflexive practice and by grounding interpretations in data extracts rather than researcher assertion. Transferability was facilitated through detailed description of the study context and participant characteristics, enabling readers to assess the relevance of findings to similar pediatric auditory rehabilitation settings.

The COREQ checklist was used as a reporting guideline to support transparency in the presentation of the study (Tong et al., 2007).

### 2.6 Ethical considerations

This study was approved by the Istanbul Medipol University Ethics Committee (decision number E-10840098-202.3.02-2003, dated March 18, 2024). The study was conducted in accordance with the principles of the Declaration of Helsinki. All participants were fully informed about the purpose and procedures of the study, and written informed consent was obtained prior to participation. Participants’ identities were anonymized during transcription, and all audio recordings were securely destroyed following data transcription and analysis. Participation was voluntary, and participants were informed of their right to withdraw from the study at any time without consequence.

## 3 Results

### 3.1 Theme 1: initial emotional response following diagnosis

Parents in both focus groups described strong emotional reactions following the diagnosis of their child’s hearing loss. However, differences emerged in how parents described their initial responses and the extent to which they engaged with the rehabilitation process in the early stages.

Parents of children with age-appropriate language development frequently described a relatively short period of shock, followed by acceptance and a focus on taking action. These parents emphasized consulting professionals, making decisions regarding amplification, and initiating auditory rehabilitation without prolonged delay.

*ŞG*: “…of course, accepting it was a process…We got used to this situation by consulting doctors or audiologists…We said okay. We were convinced right away…”

*ÇA*: “So we focused entirely on how we could solve it, we didn’t much have phase as not accepting it…then we immediately bought a behind-the-ear device and came here for aural-verbal therapy…”

In contrast, parents of children with delayed language development more often described a prolonged period of emotional distress and uncertainty following the diagnosis. Their accounts frequently included references to shock, confusion, prolonged emotional distress, and, in some cases, delays in accessing evaluation or amplification.

*HC*: “There’s a horrible nervous breakdown at first. This lasted three to four months. We were in shock for a few months.”

*YÇ*: “I swear, you can’t think of anything. You feel bad. We couldn’t collect our thoughts for a few months; it was like we were in shock.”

*TA*: “My husband’s hair turned white in a week. What you see in the movies is real.”

Across both groups, parents described the diagnosis as an emotionally challenging experience. However, accounts from parents of children with age-appropriate language development more often emphasized early acceptance and proactive engagement, whereas parents of children with delayed language development more frequently highlighted prolonged shock and delays in action.

### 3.2 Theme 2: approaches to managing the process following diagnosis

Parents in both focus groups described how they managed the period following their child’s hearing loss diagnosis and how they navigated interactions with healthcare providers, family members, and the rehabilitation system. However, differences emerged in how parents described taking responsibility for the rehabilitation process and responding to challenges encountered along the way.

Parents of children with age-appropriate language development frequently described adopting a pragmatic and solution-focused approach. Their accounts emphasized following professional guidance, maintaining emotional composure, and prioritizing their child’s needs despite external pressures or differing opinions within the family.

*ŞG*: “My mother and father said, ‘Why do you need to get a report for this child? Don’t label the child.’ But of course we did what the doctors said. We said this child has a problem and we will do what the doctors say to prevent bigger problems.”

*HK*: “We didn’t panic when we heard it. No matter what anyone said, this was the truth.”

These parents described making decisions based on professional recommendations, even when these decisions conflicted with advice or concerns expressed by extended family members.

In contrast, parents of children with delayed language development more often described difficulties navigating the post-diagnosis period. Their accounts included references to difficulty processing information, uncertainty about available services, and challenges in taking timely action.

*TA*: “We noticed it [the hearing loss] in the first 8-9 months, but since we could only find an appointment at XXXXX [name of the hospital] until a year later, we were only able to get the device when he was 2 years old.”

*YÇ*: “Yes, yes, but the thing is, he failed the test [the national newborn hearing screening test], but they didn’t tell us at XXXX hospital. We learned this when he was three. The device was fitted when he was 4.”

Several parents in this group also described challenges related to accessing information or navigating the rehabilitation system, including uncertainty about procedures, services, or responsibilities following diagnosis.

*ŞH*: “Frankly, I didn’t know about the report [for rehabilitation]. No one told us we needed one.”

Across both groups, parents described the post-diagnosis period as emotionally demanding. However, accounts from parents of children with age-appropriate language development more frequently emphasized decisive action and adherence to professional guidance, whereas parents of children with delayed language development more often highlighted emotional distress, uncertainty, and difficulties in managing the rehabilitation process.

### 3.3 Theme 3: parental perceptions of child development

Parents differed in how they described and evaluated their children’s developmental abilities, particularly in relation to language and communication. Variations emerged in the extent to which parents appeared aware of their children’s current abilities and limitations.

Parents of children with age-appropriate language development tended to describe their children’s skills in a measured and realistic manner. Their accounts reflected awareness of both strengths and areas requiring support, without portraying their children as developmentally advanced beyond their observed abilities.

In contrast, parents of children with delayed language development more frequently described their children’s language and communication skills in highly positive terms, despite the presence of language and communication difficulties identified during clinical assessment. These parents emphasized perceived progress and success, often framing their children as performing at or above the level of their peers.

*ŞH*: “The process is going well. There are good developments.”

*HC*: “His peers don’t ask many questions. I think he asks too many because we give him the chance.”

Across accounts from this group, parents’ descriptions emphasized improvement and competence, even in the presence of ongoing language development difficulties. Differences between groups therefore appeared not only in reported rehabilitation experiences, but also in how parents perceived and articulated their children’s developmental status.

In some accounts, these positively framed perceptions were accompanied by tensions or contrasting descriptions regarding children’s development, parenting practices, and the rehabilitation process. Such patterns appeared more prominently in the accounts of parents of children with delayed language development, whereas narratives from parents of children with age-appropriate language development generally appeared more stable across discussions.

Parents in this group occasionally provided contrasting descriptions within the same discussion, particularly when referring to their parenting approaches, the level of attention given to the child with hearing loss, and perceptions of the child’s developmental progress.

*ŞH*: “I let him take on responsibilities. I don’t spoil him.”

*ŞH*: “It’s very interesting, but our attention really shifts toward him.”

Similarly, some parents described delayed intervention as having a negative impact on their child’s development, while later portraying the child as developmentally advanced or comparable to peers with normal hearing.

*YÇ*: “I wish I could go back in time. I should have had a device put in when he was first born. He would be much better now.”

*YÇ*: “He is way ahead of his peers. I made sure he caught up with children with normal hearing.”

These tensions suggested variability in how some parents interpreted and communicated their children’s developmental progress across different contexts of the discussion. In comparison, accounts from parents of children with age-appropriate language development generally appeared more consistent across different aspects of the discussion.

### 3.4 Theme 4: integrating communication strategies into everyday life

Parents differed in how they described transferring communication skills practiced during rehabilitation sessions into everyday interactions with their children. Variations were evident in the extent to which parents reported creating opportunities for verbal communication beyond the clinical setting.

Parents of children with age-appropriate language development frequently described intentionally embedding communication-focused activities into daily routines. Their accounts emphasized encouraging verbal interaction during ordinary activities and extending strategies used in therapy to home and social environments.

*EÇ*: “For example, I sit her in front of the window. What do you see? Draw it and tell me. She draws and tells, makes sounds.”

*Zİ*: “We believe education continues at home. When we see a vehicle, a motorcycle, or an animal, I encourage my child to make the sound.”

In contrast, parents of children with delayed language development less frequently described deliberate efforts to generalize communication strategies to daily life. Their accounts more often focused on interactions occurring within the child’s comfort zone or on alternative communication approaches, with fewer references to encouraging verbal communication across contexts.

*ŞH*: “He usually asks questions by giving me a kiss…there isn’t really anyone at home who talks to him much.”

*HC*: “He uses sign language very well.”

Some parents also described how these interaction patterns affected family dynamics, including relationships with siblings.

HC: “His sister complains and says, ‘you love me less.’”

Across accounts, differences appeared in how parents described supporting communication outside the rehabilitation setting, with parents of children with age-appropriate language development more often emphasizing intentional generalization of skills to everyday life.

### 3.5 Theme 5: adaptation to hearing devices

Parents described the process of their children’s adaptation to hearing aids or cochlear implants as a central component of the rehabilitation journey. While initial resistance to device use was commonly reported across both groups, differences emerged in how parents described managing this process and sustaining consistent device use over time.

Parents of children with age-appropriate language development frequently emphasized persistence, routine-building, and their own determination in supporting device use. Their accounts highlighted repeated efforts to reintroduce the device, patience in the face of resistance, and the establishment of daily routines that normalized device use within the child’s life.

*HK*: “He constantly took the hearing aid out at first, but we kept putting it back in. Now, when he wakes up, he brings it to us himself and wears it all day.”

*EÇ*: “When you see the child accept the device, you feel like you’ve accomplished something.”

These parents often framed adaptation as a process requiring active parental involvement and consistency, rather than as a child-led decision.

In contrast, parents of children with delayed language development more often described adaptation as a challenging and, at times, unresolved process. Their accounts included descriptions of children resisting device use, removing or rejecting the device, and expressing discomfort. In several cases, parents emphasized the child’s preferences or reactions as determining whether the device was used consistently.

*TA*: “He was throwing the hearing aid out the window. He hid it so we would step on it.”

*HC*: “He was never into it. From the first ear mold, he didn’t want it. We told him he had to wear it, but it was very difficult.”

Across accounts, adaptation to hearing devices was described as an emotionally charged process for parents. However, differences appeared in how parents positioned themselves within this process, with parents of children with age-appropriate language development more frequently emphasizing sustained parental involvement and routine, whereas parents of children with delayed language development more often described adaptation as shaped by the child’s resistance.

### 3.6 Theme 6: parent involvement and shared parenting in the rehabilitation process

Parents described the role of fathers in the rehabilitation process in varying ways, highlighting differences in levels of involvement, support, and shared responsibility. Accounts suggested that fathers’ participation shaped both parental experiences and the organization of rehabilitation-related responsibilities within the family.

Parents of children with age-appropriate language development more frequently described fathers as actively involved and supportive. Their accounts emphasized shared responsibility, emotional support, and practical engagement in rehabilitation-related tasks.

*EÇ*: “I feel comfortable because my child’s father is very supportive. That puts my mind at ease.”

*HK*: “One of us has to take care of this child. My wife looks after our other children, so I take him to rehabilitation. I even thought about quitting my job.”

In contrast, parents of children with delayed language development more often described fathers as less involved or primarily focused on work-related responsibilities. Some accounts included references to limited support or to tensions within the family related to caregiving roles.

*ŞH*: “To be frank, our father doesn’t have much influence. He’s busy working.”

*HC*: “He isn’t much help, but at least he doesn’t get in my way.”

*TA*: “Fathers usually blame mothers, saying you couldn’t take care of them.”

Across accounts, differences appeared in how parents described fathers’ roles within the rehabilitation process. While some parents emphasized shared responsibility and support, others highlighted limited involvement or strained dynamics related to caregiving expectations.

Overall, the analysis identified a set of interrelated themes describing parents’ emotional responses, management of the rehabilitation process, perceptions of their children’s development, and everyday practices related to communication and device use. While some themes were present across both groups, differences appeared in how parents described early engagement, consistency, and involvement in rehabilitation-related activities. Parents of children with age-appropriate language development more frequently emphasized proactive engagement, structured routines, and shared caregiving responsibilities, whereas parents of children with delayed language development more often described emotional strain, variability in practices, and challenges in sustaining rehabilitation processes. These themes provide a descriptive account of parental experiences that informed subsequent interpretation.

## 4 Discussion

### 4.1 Overview of key findings

This study provides a qualitative exploration of parental attitudes, thoughts, and behaviors related to the auditory rehabilitation and language development of children with hearing loss. Consistent with prior research, the findings demonstrate that parental experiences are shaped not only by access to early identification and intervention services, but also by how parents emotionally process the diagnosis, interpret their child’s development, and integrate rehabilitation practices into everyday family life (Kurtzer-White and Luterman, 2003; Young and Tattersall, 2007).

Across themes, differences between parents of children with age-appropriate language development and those with delayed language development were evident in patterns of engagement, consistency of rehabilitation practices, perceptions of child development, and family dynamics. These findings highlight that rehabilitation outcomes are influenced by a complex interplay between clinical intervention and parental meaning-making processes.

### 4.2 Parental responses following diagnosis

The period following diagnosis emerged as a critical phase shaping parental engagement with rehabilitation. Parents who described a relatively rapid transition from emotional reaction to action reported earlier and more consistent involvement in amplification and rehabilitation processes. This finding aligns with previous research indicating that early parental acceptance and engagement are associated with more sustained intervention practices (Adams, 2011; Young and Tattersall, 2007).

In contrast, parents who described prolonged emotional distress reported greater difficulty navigating rehabilitation pathways. Emotional responses such as shock, grief, and confusion have been widely documented as normative following the identification of hearing loss (Kurtzer-White and Luterman, 2003; Moses, 1987). The present findings suggest that variability in how parents process this period may influence not only the timing of intervention, but also the consistency with which rehabilitation practices are sustained over time. At the same time, these findings should be interpreted within the broader rehabilitation context in Türkiye, where variability in family engagement with follow-up services, rehabilitation participation, healthcare navigation, and access to information may also influence intervention trajectories despite the availability of nationwide newborn hearing screening and referral systems.

### 4.3 Parental perceptions of child development and meaning-making

A salient finding of this study concerns differences in how parents perceived and articulated their children’s language and communication abilities. Parents of children with delayed language development frequently emphasized perceived progress and competence, sometimes describing their children as performing at or above peer levels despite documented developmental challenges. Similar patterns have been described in qualitative studies exploring parental meaning-making following cochlear implantation, where parents actively reframe developmental progress as a way of sustaining hope and motivation (Zaidman-Zait, 2007; Zaidman-Zait and Young, 2008).

Such interpretations should not be understood as misperceptions, but rather as adaptive coping processes through which parents manage ongoing uncertainty and emotional burden (Moses, 1987). At the same time, these perceptions may influence how parents evaluate the urgency of rehabilitation goals, underscoring the importance of sensitive, ongoing communication between professionals and families regarding developmental expectations.

### 4.4 Inconsistencies in parental accounts

Inconsistencies within parental narratives emerged primarily among parents of children with delayed language development. Parents sometimes described delayed intervention as detrimental while simultaneously portraying their child as developmentally advanced. Similar inconsistencies have been noted in the broader literature on coping and adjustment, where individuals may hold seemingly contradictory views as part of an adaptive meaning-making process (Chabrol and Callahan, 2004).

Rather than indicating disengagement, such inconsistencies may reflect parents’ attempts to reconcile clinical information with everyday observations of their child. Recognizing these patterns is important for clinicians, as they highlight the need for ongoing dialog that supports parents in integrating professional guidance with lived experience.

### 4.5 Everyday communication practices and parental involvement

The findings underscore the importance of everyday communication practices in supporting language development. Parents of children with age-appropriate language development are more frequently described actively embedding communication strategies into daily routines, extending therapeutic approaches beyond structured sessions. This observation is consistent with evidence demonstrating that parent training and family-centered intervention contribute positively to communication outcomes in children with hearing loss (Giallini et al., 2021; Lam-Cassettari et al., 2015).

In contrast, parents of children with delayed language development less frequently described systematic generalization of communication strategies to daily life. This variability suggests that rehabilitation success depends not only on service provision, but also on the extent to which parents are supported in translating therapeutic strategies into everyday interactions.

### 4.6 Hearing device use, stress, and family dynamics

Adaptation to hearing devices was described as an emotionally demanding process across families. Consistent with previous research, initial resistance to device use was common, and sustained parental involvement was required to establish consistent use (Caballero et al., 2017; Sarant and Garrard, 2014). Parents who reported greater consistency in device use often described structured routines and shared caregiving responsibilities within the family.

The role of fathers emerged as a particularly important aspect of family dynamics. Greater paternal involvement was described in families reporting more consistent rehabilitation practices, supporting earlier findings that father participation contributes to more positive communication and developmental outcomes in children with hearing loss (Calderon and Low, 1998; Hadadian and Rose, 1991). These findings highlight the value of engaging the broader family system in pediatric auditory rehabilitation.

### 4.7 Methodological considerations and limitations

This study contributes to the literature by providing an in-depth qualitative examination of parental experiences using reflexive thematic analysis. Nevertheless, several limitations should be acknowledged. The small sample size and focus group design limit transferability beyond similar clinical and sociocultural contexts. In addition, the two focus groups differed in certain demographic and contextual characteristics, including parental education, family structure, and age at diagnosis, which may have influenced parental experiences and rehabilitation-related practices. Additionally, parental accounts reflect subjective experiences and interpretations, which may not directly correspond to objective measures of rehabilitation adherence or language outcomes.

Despite these limitations, qualitative approaches offer unique insight into parental meaning-making processes that are difficult to capture through quantitative methods alone, particularly in the context of pediatric hearing loss and rehabilitation.

### 4.8 Implications for practice and future research

The findings of this study have direct implications for patient education and counseling practices in pediatric auditory rehabilitation. First, parental emotional responses following diagnosis should be routinely assessed and addressed as part of early clinical encounters. Incorporating brief, structured counseling components into standard appointments may help clinicians identify parental readiness, normalize emotional reactions, and support adaptive coping strategies.

In the clinical context of the present study, families of children with hearing loss are primarily supported by audiologists and speech-language therapists during the rehabilitation process. Routine involvement of psychosocial professionals such as psychologists or social workers is relatively limited. However, the findings of the present study suggest that parental emotional distress, coping difficulties, and variability in family dynamics may substantially influence rehabilitation engagement and everyday communication practices. From this perspective, incorporating psychosocial support services into multidisciplinary pediatric hearing rehabilitation teams may provide important benefits for both parents and children. Structured psychological counseling or family support interventions may help families process the diagnosis, manage emotional burden, and maintain more consistent engagement with rehabilitation.

Second, the results indicate that providing information about hearing devices and therapy is insufficient to ensure sustained engagement. Clinicians should adopt a more active coaching role by explicitly guiding parents on how to implement communication strategies in everyday contexts. This may include modeling parent–child interaction techniques during sessions, setting concrete home-based tasks, and providing ongoing feedback.

The findings further suggest that some families of children demonstrating delayed language development may experience difficulties recognizing or fully acknowledging ongoing communication and rehabilitation-related challenges. In several accounts, parents emphasized positive developmental interpretations despite inconsistencies in rehabilitation practices or documented language delays. These patterns may reflect adaptive coping or protective meaning-making processes rather than intentional disengagement.

From a clinical perspective, this suggests that simply increasing the intensity of services may not be sufficient for all families. Instead, clinicians may need to adopt more collaborative and reflective counseling approaches that support parental insight, expectation alignment, and gradual engagement with rehabilitation-related challenges without undermining hope or parental self-efficacy. Such approaches may help strengthen therapeutic alliance and promote more sustainable participation in rehabilitation processes.

Third, the findings highlight the need for continuous alignment between clinical expectations and parental perceptions of child development. Regular, structured discussions using clear developmental benchmarks may help reduce discrepancies between perceived and actual progress and support more realistic and goal-oriented engagement.

Finally, involving multiple caregivers, particularly fathers, in counseling and rehabilitation processes should be actively encouraged. Expanding counseling beyond a single primary caregiver may improve consistency in communication practices, device use, and overall adherence to rehabilitation recommendations.

Overall, these findings support the integration of structured, counseling-informed, and family-centered approaches into routine audiological care, emphasizing emotional support, practical skill-building, and active caregiver engagement as core components of effective rehabilitation.

## 5 Conclusion

This qualitative study highlights that parental engagement in pediatric auditory rehabilitation extends beyond access to services and is deeply shaped by how parents interpret, manage, and integrate the rehabilitation process into everyday life. Differences between parents of children with age-appropriate language development and those with delayed language development were evident across emotional responses following diagnosis, perceptions of child development, consistency of rehabilitation practices, and family dynamics.

The findings suggest that early engagement, sustained parental involvement, and the generalization of communication strategies into daily routines are closely intertwined with how parents make sense of their child’s hearing loss and rehabilitation journey. Importantly, variability in parental perceptions of child development and internal inconsistencies in parental accounts underscore that rehabilitation outcomes are influenced not only by clinical interventions, but also by parental meaning-making processes and family context.

These results emphasize the need for family-centered approaches that address parental emotional processing, expectations, and everyday practices alongside technical aspects of amplification and therapy. Supporting parents in navigating the post-diagnosis period, fostering realistic yet hopeful developmental perspectives, and encouraging consistent integration of communication strategies into daily life may contribute to more sustained engagement in rehabilitation.

Overall, this study contributes to the qualitative literature by offering an in-depth understanding of parental experiences in pediatric hearing loss and underscores the importance of considering parental perspectives as a central component of effective auditory rehabilitation.

## Data Availability

The datasets presented in this study can be found in online repositories. The names of the repository/repositories and accession number(s) can be found in the article/supplementary material.
